# Effect of Ni Doping on the Thermoelectric Properties of YbCo_2_Zn_20_

**DOI:** 10.3390/ma17081906

**Published:** 2024-04-19

**Authors:** Jorge R. Galeano-Cabral, Benny Schundelmier, Olatunde Oladehin, Keke Feng, Juan C. Ordonez, Ryan E. Baumbach, Kaya Wei

**Affiliations:** 1 FAMU-FSU College of Engineering, Florida State University, Tallahassee, FL 32310, USA; jgaleanocabral@fsu.edu (J.R.G.-C.); ordonez@eng.famu.fsu.edu (J.C.O.); 2 National High Magnetic Field Laboratory, Florida State University, Tallahassee, FL 32310, USA; bcschundelmier@fsu.edu (B.S.); ooladehin@fsu.edu (O.O.); kefeng@ucsd.edu (K.F.); rbaumbac@ucsc.edu (R.E.B.); 3 Department of Physics, Florida State University, Tallahassee, FL 32306, USA

**Keywords:** 1-2-20 compounds, thermoelectric materials, heavy-fermion materials, doping, flux growth

## Abstract

Thermoelectric devices are both solid-state heat pumps and energy generators. Having a reversible process without moving parts is of high importance for applications in remote locations or under extreme conditions. Yet, most thermoelectric devices have a rather limited energy conversion efficiency due to the natural competition between high electrical conductivity and low thermal conductivity, both being essential conditions for achieving a high energy conversion efficiency. Heavy-fermion compounds Yb*T*_2_Zn_20_ (*T* = Co, Rh, Ir) have been reported to be potential candidate materials for thermoelectric applications at low temperatures. Motivated by this result, we applied chemical substitution studies on the transition metal site in order to optimize the charge carrier concentration as well as promote more efficient phonon scatterings. Here, we present the latest investigation on the Ni-doped specimens YbCo_2−*x*_Ni_*x*_Zn_20_, where enhanced thermoelectric figure of merit values have been obtained.

## 1. Introduction

Thermal processes are a fundamental aspect of many crucial operations, ranging from water purification protocols to energy generation [1,2]. However, a persistent challenge prevails: the dissipation of valuable energy in the form of heat throughout these processes. Consequently, there has been a growing interest in waste-heat recovery systems to improve the efficiency of energy operations. Among the array of solutions, thermoelectric generators (TEGs) have emerged as promising contenders, capitalizing on the Seebeck effect to directly convert heat into electrical power. TEGs have been proposed for a wide variety of applications including waste-heat recovery from automobile engines [3,4,5], cooling of electronics [6,7,8], health monitoring [9,10], space power [11,12], and vaccine storage [13,14]. While strides have been made in making TEGs viable for real-world applications, the domain of thermoelectric technology still grapples with substantial hurdles, notably exemplified by the low values of the Seebeck coefficient, *S*, observed in the majority of thermoelectric materials. *S* can be defined as the ratio between the voltage and the corresponding temperature gradient (S=ΔV/ΔT). The figure of merit of a thermoelectric material is given by $Z=S^{2}\sigma /\kappa$, where σ and κ denote the electrical and thermal conductivities, respectively. Because the figure of merit is temperature-dependent, the primary metric for evaluating the efficiency of thermoelectric materials is $ZT=S^{2}\sigma T/\kappa$.

From the figure of merit, it is clear that to improve thermoelectric performance, it is desirable to identify materials with large electrical conductivity, low thermal conductivity, and a large Seebeck coefficient. It is worth noting that σ and κ are intrinsically linked, posing a limitation for conventional metals and semiconductors [15,16].

The electrical conductivity is related to the density of charge carriers (*n*) and their mobility (μ), and it is typically expressed as σ=neμ, where *e* is the charge of the electron. Large electrical conductivity reduces Joule heating. The mobility is given by $\mu =e\tau /m_{e}$ where $m_{e}$ is the effective mass and τ is the mean scattering time between collisions for the carriers. In an intrinsic semiconductor, the two types of charge carriers, with densities *n* and *p* for electrons and holes, respectively, move through the lattice with mobilities $\mu_{e}$ and $\mu_{h}$, respectively. In this case, the conductivity occurs through the contributions of both holes ($\sigma_{h}$) and electrons ($\sigma_{e}$), as follows: $\sigma =\sigma_{e}+\sigma_{h}=ne\mu_{e}+pe\mu_{h}$. Since electrons and holes are fermions, the probability of their occupation of a given energy state is determined by Fermi–Dirac statistics. The electrical conductivity is typically optimized as a function of the carrier concentration through extrinsic doping to produce *P*- and *N*-type materials [17].

The high absolute value of the Seebeck coefficient, *S*, would result in a high conversion of heat to electrical power, or electrical power to cooling performance. The sign of *S* indicates the majority of the charge carriers. For a positive value, the holes are the major carriers, and a negative value means the electrons dominate. The change of the sign of *S* with respect to *T* is an indication of a two-band model in the material, a competition between electrons and holes for dominating the charge transport [18]. *S* for different carrier types (e.g., an intrinsic semiconductor) is determined by the partial Seebeck coefficient of the electron ($S_{e}$) and holes ($S_{h}$) through the following relation: $S=\left(S_{e}\sigma_{e}+S_{h}\sigma_{h}\right)/\left(\sigma_{e}+\sigma_{h}\right)$. Extrinsic conduction of the appropriate carrier type is possible by doping the material with either electrons or holes. Since *S* will be lower than that of either of the individual contributions, good thermoelectric materials have a band gap large enough to effectively minimize the minority carrier contributions [19]. Possible mechanisms to increase *S* include phonon drag, heavy-fermion materials, Kondo systems, and materials that exhibit phase transitions [20].

Low thermal conductivity values minimize the transfer of heat through the material. This transfer of heat could be done by electrons or via quantized vibrations of the lattice (phonons), such that $\kappa =\kappa_{L}+\kappa_{E}$, where $\kappa_{L}$ and $\kappa_{E}$ are the lattice and electronic contributions, respectively. κ and σ are connected through the Wiedmann–Franz relationship [21,22], $\kappa_{E}=\sigma L_{0}T$, where the Lorentz number $L_{0}=2.44\times 10^{-8}\;W\Omega /K^{2}$. The ratio between σ and $\kappa_{E}$ is constant at a given temperature, which means that a more efficient way to reduce the total thermal conductivity would be to decrease the lattice contribution $\kappa_{L}$. This could be achieved by scattering phonons in a wide frequency range, which is possible through a variety of methods, such as tuning the mass fluctuation phonon scatterings in ternary and quaternary compounds [19].

For metals or degenerate semiconductors, the relationship between the charge carrier density, *n*, and Seebeck coefficient, *S*, can be expressed as

(1) $$S=\frac{8\pi^{2}\kappa_{B}^{2}}{3eh^{2}}m^{*}T{\left(\frac{\pi }{3n}\right)}^{2/3}$$

where *n* is the density of the charge carriers and $m^{*}$ is the effective mass of the carrier. Here, another conflict arises with the effective mass of the charge carrier, where larger effective masses result in a higher Seebeck coefficient but lower electrical conductivity. $m^{*}$ in Equation (1) is related to the curvature of the Fermi surface, and it increases when there is a large density of states close to the Fermi surface; however, heavy carriers will exhibit reduced velocities, resulting in diminished mobilities and subsequently leading to decreased electrical conductivity [23]. Therefore, a balance needs to be found when considering the effective mass of the predominant charge carrier in order to create a trade-off between a large effective mass and high mobility.

Significant progress has been made in the development of certain intermetallic materials for thermoelectric applications. Intermetallic compounds composed of rare-earth and transition metals have earned considerable attention due to their versatile properties, which manifest in exotic magnetism and strongly correlated electronic behaviors [24,25,26,27]. This versatility has led to the emergence of intriguing thermoelectric characteristics in specific intermetallic materials [28,29]. Notably, increased focus has been directed toward materials incorporating Yb, which can undergo hybridization with conduction electrons, resulting in what is known as heavy-fermion behavior. Compounds exhibiting a combination of these intriguing characteristics have been a focus of interest among scientists for quite some time. For example, the *RT*_2_Zn_20_ (*R* = rare earth, *T* = transition metal) family of materials, as discovered by Nasch et al. [30], has been the subject of study for over 25 years. Among its variants, the Yb analog has shown promise for low-temperature thermoelectric applications. Specifically, the heavy-fermion compounds Yb*T*_2_Zn_20_ (*T* = Co, Rh, Ir) exhibit favorable *S* and *ZT* values at lower temperatures [31,32]. Among the three specimens, YbIr_2_Zn_20_ has emerged as the most promising in terms of thermoelectric performance, exhibiting a peak figure of merit (*ZT*) of approximately 0.07 at 35 K. In contrast, the Co and Rh analogs demonstrate lower *ZT* values of 0.0024 and 0.025, respectively. Further studies have revealed the potential for enhancing the thermoelectric properties of the Ir variant by incorporating other *f*-electron elements into the Yb site. Notably, the compound ${\mathrm{Yb}}_{0.87}{\mathrm{Ce}}_{0.08}{\mathrm{Sm}}_{0.05}{\mathrm{Ir}}_{2}{\mathrm{Zn}}_{20}$ [33] achieved a ZT value of 0.076 at 18.63 K, positioning it as the second-best performer, trailing only behind the state-of-the-art solid solutions ${\mathrm{Bi}}_{1-x}$${\mathrm{Sb}}_{x}$ with ZT≈ 0.15 at 35 K [34,35]. This achievement significantly surpasses other well-known cryogenic thermoelectric materials such as FeSb_2_ [36], Bi_0.5_Sb_1.5_Te_3_, Bi_2_Te_3_, CsBi_4_Te_6_ [37], and YbAl_3_ [38], all of which exhibit ZT values below 0.03 at temperatures below 50 K. However, a notable challenge in achieving such enhancements lies in synthesizing a doping series, as not all substitutions on the Yb site yield the desired 1-2-20 structure, particularly those involving triple fillers.

It is also important to note that among the three specimens reported by Wei et al. [32], the Co version is the cheapest and most abundant variant, which is an important factor for practical applications. Rhodium (Rh) and Iridium (Ir) constitute only 0.0001 and 0.001 ppm of the Earth’s crust, respectively, which make them exceedingly rare elements compared to the 29 ppm of Cobalt (Co) [39]. Additionally, Co, unlike Rh and Ir, is part of the 35 mineral commodities identified as critical. Critical materials are used in many products important to the U.S. economy and national security [40]. Therefore, it is unlikely that these materials would not be available for research and development. The U.S. Department of Energy (DOE) assesses material criticality based on its importance to a range of energy technologies and the potential for supply risk. Co, for instance, is a battery-critical material used in electric vehicles and grid storage (in the battery cathode, Co enables high energy density and thermal stability) [41], and it is also used in the production of permanent magnets for high-temperature applications, such as SmCo_5_ and YCo_5_ [42]. Consequently, in our investigation, we chose to prioritize the YbCo_2_Zn_20_ specimen. We have decided to dope the Co site with Ni, hoping to optimize the charge carrier concentration and promote a stronger hybridization between the *f*- and conduction electrons. This strategy could lead to an enhancement in the Seebeck coefficient and, consequently, a better ZT. Elevating the performance of the Co variant to match that of the Ir counterpart could unlock significant opportunities within the industry.

For this study, we synthesized six single-crystalline samples through the molten metal flux growth method. In Section 3.2, we survey the thermoelectric properties in this doping series to establish the impact of Ni substitution on the Co site. In Section 3.3, we examine the impact of chemical substitution on the magnetic properties and heat capacity, which provides insights into the hybridization between the *f*- and conduction electron states. In Section 3.4, a Hall-effect analysis is performed to determine the connection between Ni doping, the charge carrier concentration, and the enhanced thermoelectric values. Collectively, these measurements elucidate the parameters governing the behavior of YbCo_2_Zn_20_, offering valuable insights for optimizing its thermoelectric properties.

## 2. Materials and Methods

### 2.1. Sample Synthesis

**Figure 1:**
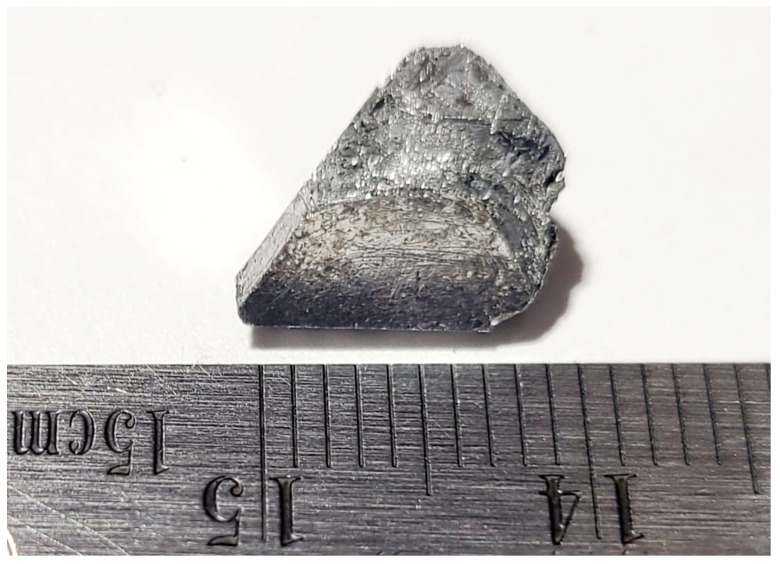
A single crystal of YbCo_1.86_Ni_0.14_Zn_20_ synthesized using the flux growth method.

Single-crystalline samples were synthesized from a Zn-rich self-flux, employing the growth procedures outlined by Torikachvili et al. [43]. Elements with purities greater than 99.9% were loaded into 2 mL Canfield alumina crucibles [44] with a molar ratio of 1(Yb):2(Co, Ni):60(Zn). The crucibles were sealed under vacuum in 2 mm thick quartz tubes, heated to 1050 °C at a rate of 50 °C/h, and held at that temperature for 72 h. Next, the ampules were cooled down to 700 °C at a rate of 2 °C/h and held at that temperature for another 72 h. Finally, at 700 °C, the excess flux was removed by centrifuging, after which crystals with dimensions of several millimeters were collected. Figure 1 shows the as-synthesized YbCo_1.86_Ni_0.14_Zn_20_ single crystal.

### 2.2. Sample Characterization

In the realm of materials science and analytical chemistry, energy-dispersive X-ray spectroscopy (EDS or EDX) has emerged as a powerful tool, playing a pivotal role in the characterization and examination of diverse materials at the microscale. As an integral component of electron microscopy, EDS offers a comprehensive understanding of the elemental composition within a sample, enabling researchers to delve into the intricacies of material properties with unparalleled precision. The process involves the detection and quantification of characteristic X-rays emitted by a specimen when bombarded with a focused electron beam. In preparation for an EDS measurement, specimens are affixed to an aluminum holder using carbon tape to secure them in place. The sample holder is then introduced into a scanning electron microscope (SEM) equipped with EDS capabilities. After achieving a vacuum environment and resolving the sample image, three to five spots, each a few microns in diameter, are selected as focal points for the electron beam on each sample. The selection of multiple targets serves the purpose of evaluating the uniformity of the entire sample.

**Figure 2:**
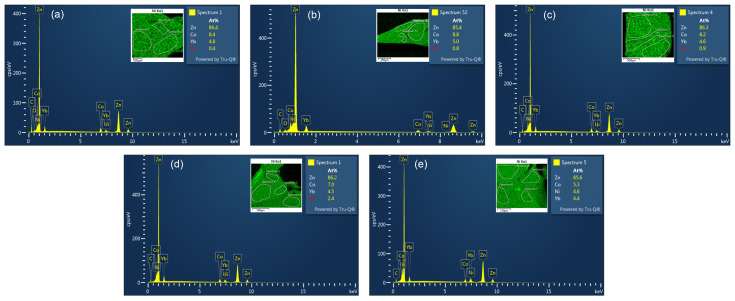
EDS spectrum of (**a**) *x* = 0.1, (**b**) *x* = 0.14, (**c**) *x* = 0.2, (**d**) *x* = 0.5, and (**e**) *x* = 1 for ${\mathrm{YbCo}}_{2-x}$${\mathrm{Ni}}_{x}$Zn_20_. The insets show the elemental mapping of Ni as an example and the atomic percentages of all elements detected in the selected spectrum.

For this study, the elemental analysis is performed using an FEI NOVA 400 nanoSEM scanning electron microscope [Field Electron and Ion (FEI) Company, Hillsboro, OR, USA]. The Nova NanoSEM is an ultra-high-resolution Low-Vacuum Schottky Field-Emission Scanning Electron Microscope (FEG-SEM). The instrument combines a field-emission electron source, an oil-free vacuum system, a magnetic immersion final lens, and a heated objective aperture [45]. The device is equipped with energy-dispersive X-ray spectroscopy (EDS or EDX) capabilities using an Oxford 100 mm^2^ UltimMax SDD (silicon drift detector) X-ray detector [Oxford Instruments plc., Abingdon, UK]. In Figure 2, the EDS spectrum of a representative focal point for all the doped samples is displayed. The insets provide the elemental mapping of Ni and the atomic percentages of all elements detected in the selected spectrum. To ensure accuracy, the values of *x* for the doping series were derived by averaging data from multiple focal points for each sample and subsequently normalizing them based on the total number of atoms in the compound formula, which is 23 for 1-2-20 compounds.

The crystal structure and unit cell parameters were characterized by powder X-ray diffraction (pXRD) using a Rigaku SmartLab SE X-ray diffractometer [Rigaku Corporation, Tokyo, Japan] with a Cu Kα source. Temperature-dependent magnetic susceptibility, χ(T), and isothermal magnetization, M(H), measurements were performed for single crystals using a VSM SQUID Magnetometer from Quantum Design, model MPMS-3 [Quantum Design Inc, San Diego, CA, USA]. M(H) measurements were taken at a constant temperature of *T* = 1.8 K under applied magnetic fields, *H*, from −7 T to 7 T. χ(T) measurements were taken under a constant magnetic field of *H* = 0.1 T from 1.8 K to 300 K. For both cases, single crystals were mounted such that the magnetic field was parallel to the (111) planes. Heat capacity ($C_{p}$) was measured from 1.8 K to 250 K using a Quantum Design Physical Property Measurement System (PPMS), model 6000 [Quantum Design Inc, San Diego, CA, USA]. The temperature-dependent Seebeck coefficient, *S*, thermal conductivity, κ, and electrical resistivity, ρ, were measured using the PPMS Thermal Transport Option (TTO). For these measurements, single crystals were cut into rectangular bars with a general dimension of 7 mm by 2 mm by 1 mm. All surfaces were polished to reduce surface scattering. TTO measurements were performed from 1.8 K to 400 K, obtaining ρ, κ, and *S* simultaneously as a function of *T* to assess both ZT and the power factor, $PF=S^{2}\sigma$. Both the $C_{p}$ and the TTO measurements were performed under high vacuum (≈$10^{-4}$ Torr), with no magnetic field being applied to the specimens. Magnetic field-dependent Hall resistance measurements were conducted at T=1.9 K across a range of magnetic fields from H=−9 T to H=9 T. Subsequently, the density of the charge carriers was determined for each sample. These measurements were performed on single crystals polished to a thickness of less than 1 mm.

## 3. Results and Discussion

### 3.1. Structural Characterization

**Figure 3:**
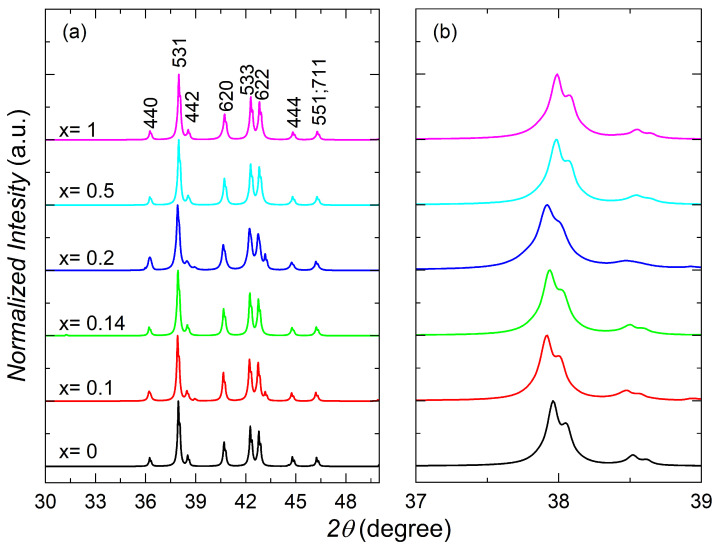
(**a**) X-ray diffraction peaks and (**b**) zoomed-in view of the highest intensity peak for ${\mathrm{YbCo}}_{2-x}$${\mathrm{Ni}}_{x}$Zn_20_. The data were obtained through powder X-ray diffraction. Miller indices are included for reference.

${\mathrm{YbCo}}_{2-x}$${\mathrm{Ni}}_{x}$Zn_20_ crystalizes in the same space group, Fd-3*m* (#227), as the other 1-2-20 compounds [46,47,48,49]. The X-ray diffraction peaks from 2θ = 30° to 50° are shown in Figure 3a, and the zoomed-in peaks from 37° to 39° are shown in Figure 3b. The peak positions across the doping series only show slight to negligible shifts. This is as expected since the atomic radii of Ni and Co are very similar [50], suggesting that this type of chemical substitution has a minimal impact on the lattice constant.

### 3.2. Electrical Transport and Thermoelectric Properties

**Figure 4:**
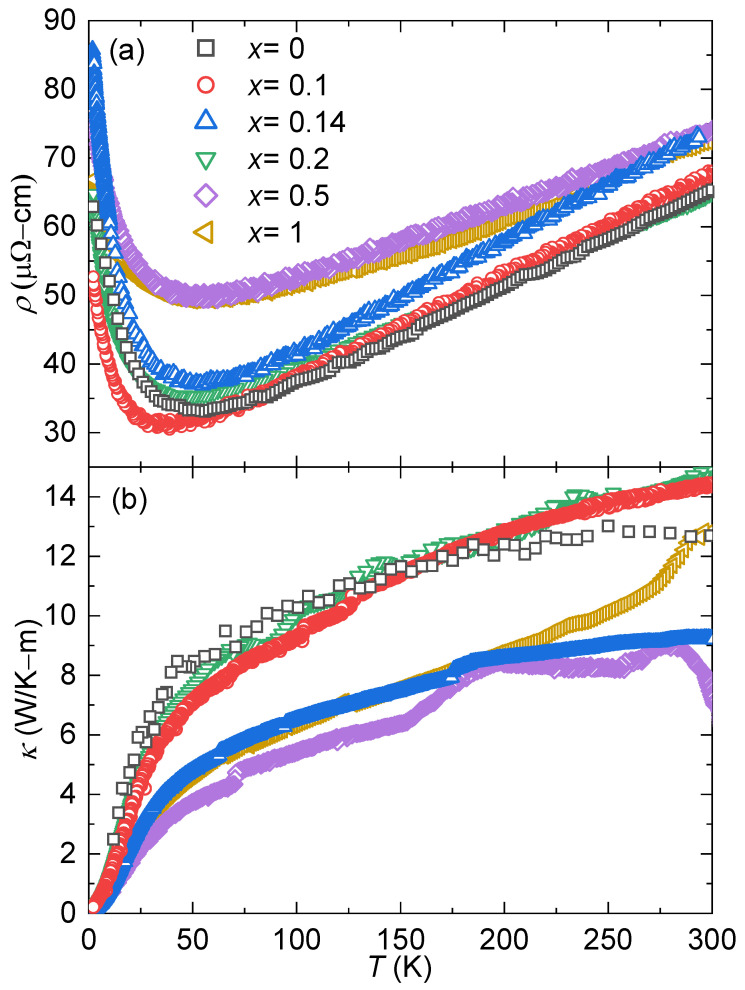
(**a**) Temperature-dependent electrical resistivity, ρ(T), and (**b**) thermal conductivity, κ(T), for ${\mathrm{YbCo}}_{2-x}$${\mathrm{Ni}}_{x}$Zn_20_.

Figure 4a shows the temperature-dependent electrical resistivities ρ(T) of all specimens of ${\mathrm{YbCo}}_{2-x}$${\mathrm{Ni}}_{x}$Zn_20_. All the samples show similar behavior, where ρ decreases with increasing temperature for 1.8 K <T< 50 K, and a broad minimum is observed. At high temperatures, T>50 K, the specimens exhibit metallic behavior, where ρ increases with increasing temperature. This behavior is consistent with earlier reports, where it was also shown that the parent compound (YbCo_2_Zn_20_) exhibited a coherence peak, typical for Kondo lattice systems [51,52,53], below 1.8 K and a sudden drop around 20 mK [43]. It can be seen that a small amount of Ni in the system does not significantly affect the behavior of the electrical resistivity; however, for specimens *x* = 0.5 and *x* = 1 (25% and 50% Ni on the Co site, respectively), the value of the minima increases from ρ≈ 30 μΩcm to ρ≈ 50 μΩcm (see Table 1). Figure 4b illustrates the thermal conductivity values for all synthesized specimens. Notably, specimens exhibiting lower thermal conductivity values also demonstrate higher electrical resistivity, except for x=0.14. Considering that all the specimens are mostly Zn, the lattice thermal conductivity values ($\kappa_{L}$) are expected to be similar across the doping series.

**Figure 5:**
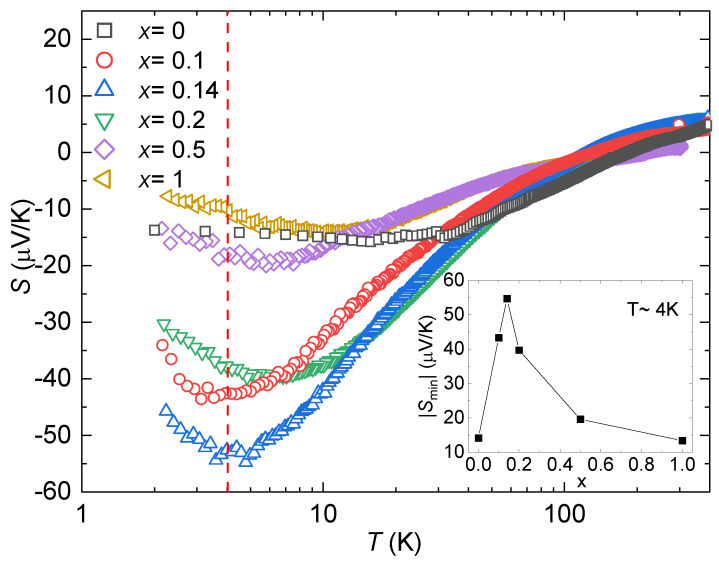
Seebeck coefficient, S(T), for ${\mathrm{YbCo}}_{2-x}$${\mathrm{Ni}}_{x}$Zn_20_. The dashed red line indicates T=4 K. The inset shows the absolute value of the Seebeck coefficient’s minima as a function of Ni concentration. Note a logarithmic temperature scale.

Looking at the Seebeck coefficient *S* (Figure 5), we can see that |S| for all the samples increases with decreasing temperature and peaks around T≈4 K, then decreases as T→0 K. This peak is more prominent in the Ni-containing samples. The inset in Figure 5 shows the absolute value of the peak as a function of the Ni content (values listed in Table 1), where an optimum content of Ni can be observed between *x* = 0.1 and *x* = 0.2 with a significant improvement in *S* compared to the parent compound. While YbCo_2_Zn_20_ has been reported to have a value of |S|≈15μV/K [32], introducing 7% of Ni in the system (*x* = 0.14) enhanced that value to |S|≈55μV/K.

**Figure 6:**
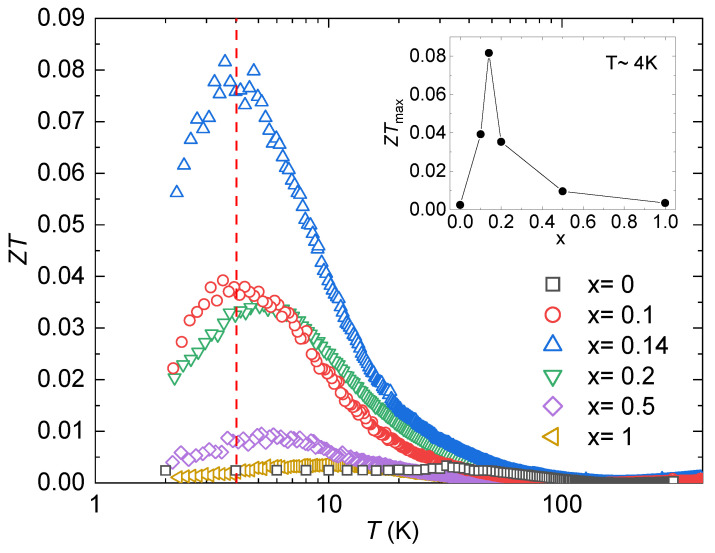
Dimensionless figure of merit, ZT(T), for ${\mathrm{YbCo}}_{2-x}$${\mathrm{Ni}}_{x}$Zn_20_. The dashed red line indicates T=4 K. The inset shows the value of the figure of merit’s maxima as a function of the Ni concentration. Note the logarithmic temperature scale.

Figure 6 shows the dimensionless figure of merit, $ZT=S^{2}T/\left(\rho \kappa \right)$. Similar to *S*, all the samples present a peak at T≈4 K, and an optimum Ni concentration can be observed (see inset in Figure 6). The values of the peaks are also listed in Table 1. The specimen that presents the highest value is YbCo_1.86_Ni_0.14_Zn_20_, with a maximum value of ZT=0.082 at 3.6 K. This value represents a remarkable enhancement compared to the parent compound YbCo_2_Zn_20_ with ZT≈0.001 at 4 K and the highest value of ZT≈0.003 at T≈ 35 K. Compared to the Ir analogs, it also represents an important improvement in ZT since the highest reported value is 0.076 at 18.63 K for ${\mathrm{Yb}}_{0.87}{\mathrm{Ce}}_{0.08}{\mathrm{Sm}}_{0.05}{\mathrm{Ir}}_{2}{\mathrm{Zn}}_{20}$ [33].

### 3.3. Magnetic and Heat Capacity Properties

**Figure 7:**
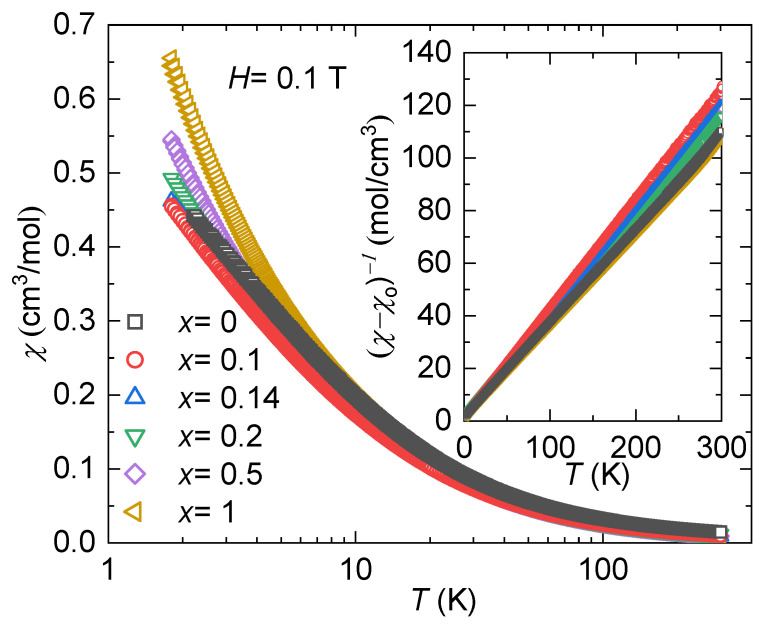
Temperature-dependent magnetic susceptibility, χ(T), with an applied magnetic field of *H* = 0.1 T for ${\mathrm{YbCo}}_{2-x}$${\mathrm{Ni}}_{x}$Zn_20_. The inset shows the inverse of the magnetic susceptibility, where an additional term $\chi_{0}$ is needed for the Curie–Weiss fit. Note the logarithmic temperature scale for χ(T).

Introducing Ni into the system is a natural way to adjust the carrier concentration since we are changing the number of electrons in the system. However, this type of doping can also affect the hybridization strength by altering the interactions between the conduction electrons and the *f*-electrons from the Yb. To investigate this effect, temperature-dependent magnetic susceptibility was measured from 1.8 K to 300 K with an applied magnetic field of *H* = 0.1 T. Figure 7 shows the magnetic susceptibility, χ(T), for all specimens for 1.8 K < *T* < 300 K. At the base temperature, the strength of the magnetic exchange interaction follows the doping series. The inset in Figure 7 shows the inverse of the magnetic susceptibility (1/χ), where a temperature-independent contribution to the susceptibility, $\chi_{0}$, is considered. This additional term could have originated from the sample holder core diamagnetism, Pauli paramagnetism, or van Vleck paramagnetism [54,55,56]. Table 1 shows the $\chi_{0}$ values used for all the samples. By taking this behavior into account, a modified form of the Curie–Weiss law (Equation (2)) is applied to fit the data at high temperatures (200 K < *T* < 300 K).

(2) $$\chi \left(T\right)=\frac{C}{T-\theta_{\mathrm{CW}}}+\chi_{0}\left(T\right)$$

where *C* represents the Curie constant and $\theta_{\mathrm{CW}}$ represents the Curie–Weiss temperature. The Curie constant, *C*, correlates directly with the number of unpaired electrons, and it enables the calculation of the effective magnetic moment per ion, measured in units of Bohr magnetons, $\mu_{B}$, through the expression $\mu_{\mathrm{eff}}=2.82\sqrt{C/n}$, where *n* represents the number of magnetic elements per formula unit [57]. Table 1 shows the values of $\mu_{\mathrm{eff}}$ and $\theta_{\mathrm{CW}}$ for all the samples. It is seen that the values of $\mu_{\mathrm{eff}}$ are in agreement with the Hund’s rules moment of $\mu_{\mathrm{eff}}=4.5\mu_{B}$ for Yb^3+^, with expected experimental values between 4.4 and 4.9 $\mu_{B}$ [58]. This indicates that the Yb remains trivalent for all substitutions. Regarding $\theta_{\mathrm{CW}}$, the parent compound indicates ferromagnetic interactions, and adding a small amount of Ni (x=0.1) switches it to antiferromagnetic interactions. However, for x=0.14 and x=1, $\theta_{\mathrm{CW}}>0$, implying that the molecular field aligns again with the external field, similar to the parent compound. It is crucial to emphasize that in the majority of rare-earth magnets, $\theta_{\mathrm{CW}}$ tends to overestimate the interaction strength. This occurs because high-temperature fittings incorporate contributions from thermally populated crystal field levels, which are absent at the lower temperatures where interactions become significant [59].

**Figure 8:**
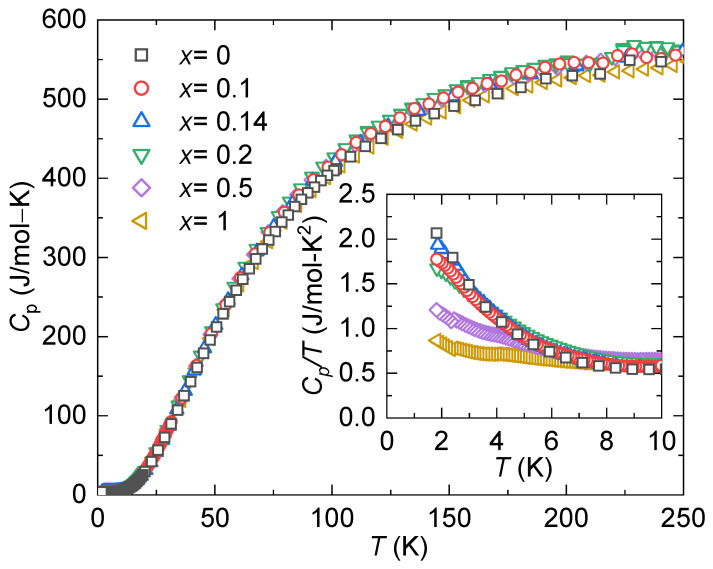
Temperature-dependent heat capacity, $C_{p}\left(T\right)$, for ${\mathrm{YbCo}}_{2-x}$${\mathrm{Ni}}_{x}$Zn_20_. The inset shows the low-temperature behavior as $C_{p}/T$ vs. *T*.

Measurements of heat capacity (shown in Figure 8) offer additional understanding regarding the development of electronic hybridization and lattice behavior. For this purpose, the heat capacity data were fitted using a modified lattice-Debye model, where the electronic contribution is included, as expressed in Equation (3) [60]:

(3) $$C_{p}=\gamma T+C_{D}{\left(\frac{T}{\theta_{D}}\right)}^{3}\int_{0}^{\theta_{D}/T}\frac{x^{4}e^{x}}{{\left(e^{x}-1\right)}^{2}}dx$$

where $x=\hbar \omega /\kappa_{B}T$, ω is the Debye frequency, *ℏ* is the Planck’s constant, $\kappa_{B}$ is the Boltzmann constant, $\theta_{D}$ is the Debye temperature, γ represents the Sommerfeld coefficient, and $C_{D}$ is a constant containing the number of oscillators and degrees of freedom.

The electronic contribution to the heat capacity, denoted by γ, exhibits enhancement between x=0 and x=0.2, followed by a subsequent decrease in value for higher concentrations of Ni (refer to Table 1). This suggests that the effective mass of the system is only enhanced for light doping. The contributions from the lattice dynamics to $C_{p}$ can be elucidated through $\theta_{D}$, also provided in Table 1. It is notable that $\theta_{D}$ shows insensitivity to doping, maintaining an approximate overall value of $\theta_{D}\approx 240$ K across all specimens, which aligns well with previously reported values for similar compounds [32,33].

### 3.4. Hall Effect

**Figure 9:**
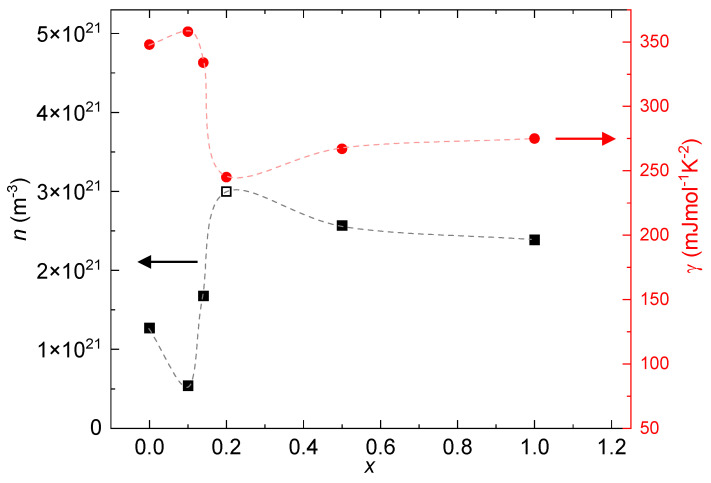
Black squares represent the density of the charge carriers *n* as a function of the Ni concentration *x* at a temperature of *T*= 1.9 K (left y-axis). The open symbol (x=0.2) represents holes as the majority of charge carriers, and the filled symbols (*x* = 0, 0.14, 0.2, 0.5, and 1) represent electrons as the majority of charge carriers. Red circles represent the Sommerfeld coefficient γ as a function of *x* (right y-axis).

The doping range where γ displays enhancement coincides with improvements in *S* and ZT. To delve deeper into this relationship, Hall-effect measurements and analysis were conducted to gain insight into the impact of the charge carrier density on the system and its role in enhancing ZT. To obtain the density of the charge carriers, *n*, the voltage across a sample (Hall voltage $V_{H}$) with thickness *t* is measured, which is related to the current $I_{x}$ and the magnetic field $B_{z}$ through Equation (4), as follows [61]:

(4) $$V_{H}=-\left(\frac{1}{nq}\right)\frac{I_{x}B_{z}}{t}$$

where *q* is the elementary charge in coulombs. Figure 9 illustrates the charge carrier concentration (*n*) for all specimens (black squares), with open symbols representing hole-dominant and filled symbols representing electron-dominant. Notably, it can be observed that *n* follows the doping series and saturates at x>0.2. Additionally, there appears to be a competitive balance between electrons and holes across the specimens since for x=0.2, the majority of the charge carriers are holes despite the introduction of more electrons into the system through the addition of Ni. Furthermore, these findings underscore the sensitivity of the system, particularly its thermoelectric properties, to Ni doping. It highlights the tunability of key thermoelectric parameters (γ, *S*, and ZT) through the strategic introduction of Ni as a dopant. Figure 9 also illustrates the relationship between γ(x) (red circles) and *n* for comparative analysis, revealing a discernible correlation between the two variables. Remarkably, both variables exhibit critical points within the range of x=0.1 to x=0.2, with tendencies toward saturation observed for x>0.2.

Through Hall-effect measurements, our analysis of the electrical resistivity and Seebeck coefficient data unveils that the optimal charge carrier concentration for this material to attain its peak thermoelectric performance is approximately $n\approx 1.68\times 10^{21}\;m^{-3}$. Regarding heat capacity, we observe a subtle influence of Ni doping on the average lattice behavior, while the electronic contribution to heat capacity indicates an improvement of the Sommerfeld coefficient (γ) within the same region where enhancements in the Seebeck coefficient (*S*) and the figure of merit (ZT) are observed (0.1 < *x* < 0.2). The Hall-effect data further highlight the correlation between optimizing the density of the charge carriers and achieving better thermoelectric properties. More specifically, there is a clear correlation between n(x) and γ(x), where the doping concentrations characterized with lower *n* values exhibit higher γ values, and vice versa (Figure 9).

## 4. Conclusions

We examined the effect of Ni doping on the thermoelectric properties of YbCo_2_Zn_20_ by synthesizing single crystals of ${\mathrm{YbCo}}_{2-x}$${\mathrm{Ni}}_{x}$Zn_20_ using the molten flux growth method. A thorough investigation was conducted on the electrical, magnetic, thermal, and thermoelectric properties of all six specimens. Our findings collectively suggest the potential for enhancing the thermoelectric properties of YbCo_2_Zn_20_ (ZT=0.0024 at 35 K) through slight Ni doping. Notably, the specimen YbCo_1.86_Ni_0.14_Zn_20_ exhibits the highest ZT, reaching a value of 0.082 at 3.6 K. We want to emphasize that substituting just 7% Ni on the Co site significantly elevates ZT, rendering it comparable to that of YbIr_2_Zn_20_, which is the specimen that presents the highest value of the figure of merit (ZT=0.07 at 35 K) among the heavy-fermion compounds Yb$T_{2}$Zn_20_ (*T* = Co, Rh, Ir) [32]. Additionally, the highest value of ZT achieved in this study is above the reported value of ${\mathrm{Yb}}_{0.87}{\mathrm{Ce}}_{0.08}{\mathrm{Sm}}_{0.05}{\mathrm{Ir}}_{2}{\mathrm{Zn}}_{20}$ (ZT = 0.076 at 18.63 K) [33], which has been reported to be the second-best thermoelectric material at *T*< 50 K among all known materials, trailing only behind the state-of-the-art solid solutions ${\mathrm{Bi}}_{1-x}$${\mathrm{Sb}}_{x}$ with ZT≈ 0.15 at 35 K [34,35]. These results underscore the feasibility of utilizing these low-temperature thermoelectric materials in practical device fabrication. One particularly promising application lies in cryogenic cooling, traditionally reliant on liquid nitrogen, which necessitates frequent and costly refilling. In contrast, leveraging thermoelectric coolers offers a compelling alternative, promising reduced costs and maintenance requirements [62,63]. This highlights a tangible advantage in transitioning toward thermoelectric-based solutions for cryogenic cooling applications.

## Figures and Tables

**Table 1:** Minimum resistivity values, $\rho_{min}$, absolute value of the Seebeck coefficient minima, $|S_{\min}|$, dimensionless figure of merit maxima, $ZT_{\max}$, magnetization parameters from Curie–Weiss fit ($\chi_{0}$, $\mu_{\mathrm{eff}}$, and $\theta_{\mathrm{CW}}$), and fitting parameters for $C_{p}$ (γ and $\theta_{D}$) of ${\mathrm{YbCo}}_{2-x}$${\mathrm{Ni}}_{x}$Zn_20_.

x	$\rho_{\min}$ [μΩcm]	$|S_{\min}|$ [μV/K]	${\mathrm{ZT}}_{\max}$	$\chi_{0}$ [${\mathrm{cm}}^{3}/\mathrm{mol}$]	$\mu_{\mathrm{eff}}$ [$\mu_{B}$]	$\theta_{\mathrm{CW}}$ [K]	γ [$\mathrm{mJ}/({\mathrm{molK}}^{2})$]	$\theta_{D}$ [K]
0	33.09	14.15	0.0024	0.005	4.63	4.60	348	244
0.1	30.48	43.35	0.0392	0.002	4.39	−5.74	358	237
0.14	36.93	54.77	0.0816	0.001	4.40	1.05	334	243
0.2	35.23	39.72	0.0353	0.003	4.57	−2.59	245	246
0.5	49.29	19.62	0.0095	0.001	4.55	−9.34	267	244
1	49.17	13.41	0.0034	0.001	4.69	2.34	275	243

## Data Availability

Data are contained within the article.
